# The role of conspiracy mindset in reducing support for child vaccination for COVID-19 in the United States

**DOI:** 10.3389/fpsyg.2023.1175571

**Published:** 2023-06-13

**Authors:** Daniel Romer, Kathleen H. Jamieson

**Affiliations:** Annenberg Public Policy Center, University of Pennsylvania, Philadelphia, PA, United States

**Keywords:** conspiracy theories, vaccination hesitancy, COVID-19, conspiracy mindset, children

## Abstract

**Introduction:**

We have previously proposed and tested a model that predicts reluctance to vaccinate against COVID-19 in the US from embrace of a conspiracy mindset that distrusts the federal health agencies of the US government and regards their intentions as malevolent. In this study, we tested the model’s ability to predict adult support for COVID vaccination of children ages 5–11 after the vaccine was approved for this age group.

**Methods:**

Relying on a national panel that was established in April 2021 (*N* = 1941) and followed until March of 2022, we examined the relation between conspiratorial thinking measured at baseline and belief in misinformation and conspiracies about COVID vaccines, trust in various health authorities, perceived risk of COVID to children, and belief in conspiracy theories about the pandemic’s origin and impact. In addition, we tested a structural equation model (SEM) in which conspiracy mindset predicted adult support for childhood vaccination for COVID in January and March of 2022 as well as the adults own vaccination status and their willingness to recommend vaccinating children against measles, mumps and rubella (MMR).

**Results:**

The model accounted for 76% of the variance in support for childhood vaccination for COVID-19; the relation between the mindset and support for vaccination was entirely mediated by baseline assessments of misinformation, trust, risk, and acceptance of pandemic conspiracy theories.

**Discussion:**

The SEM replicated the prior test of the model, indicating that a conspiracy mindset present among at least 17% of the panel underlies their resistance to vaccinate both themselves and children. Efforts to counteract the mindset will likely require the intervention of trusted spokespersons who can overcome the skepticism inherent in conspiratorial thinking about the government and its health-related agencies’ recommendations for a particular vaccine.

## Introduction

Conspiracy theories typically attribute the causes of crises and social upheavals to the secret workings of powerful agents who control events favorable to their interests rather than to the public (e.g., Uscinski, 2018; Douglas et al., 2019). Conspiracy belief is a judgment that it is probable that “an actor or group of actors … is secretly working to produce an unlawful or harmful outcome for others in society” (Albarracin et al., 2021). Some people are more disposed than others to see events in conspiratorial terms, a generalized attitude called conspiracist ideation (Brotherton et al., 2013), a conspiracy mindset (Oana and Bojar, 2023) or conspiracy mentality (Bruder et al., 2013; Imhoff and Bruder, 2014; Moscovici, 2020). Whether the prevalence of conspiracy mentality has remained stable over time (Uscinski et al., 2022) or can intensify for some people is a matter of ongoing study (Granados Samayoa et al., 2022; Romer and Jamieson, 2022).

Conspiracy theories about COVID-19 and vaccination against it have circulated throughout the COVID-19 pandemic. Among other things, these theories have alleged that the US government created the virus (Jamieson and Albarracín, 2020), 5G towers were contributing to its spread (Bruns et al., 2020), the CDC exaggerated the severity of SARS-Cov-2 to undermine the Trump presidency (Jamieson and Albarracín, 2020), and governmental efforts to quell the spread of SARS-Cov-2 are driven by malign motives (Romer and Jamieson, 2020; Jamieson et al., 2021; Tsamakis et al., 2022; van Prooijen et al., 2022; Pilch et al., 2023). The acceptance of such conspiracy theories has been especially problematic because of their association with vaccination hesitancy.

We recently proposed and tested a model that focused on evidence of an individual’s conspiratorial mindset as a powerful pre-pandemic precursor to the acceptance both of conspiracy theories about the pandemic and of misinformation about the safety and efficacy of COVID-19 vaccines (Romer and Jamieson, 2022). Research has consistently shown that people with a conspiracy mindset about the workings of government are more likely to accept a wide range of conspiracy theories (Lewandowsky et al., 2013; Hornsey et al., 2018; Dyrendal et al., 2021; Enders et al., 2021), including those associated with the pandemic (Jamieson et al., 2021; Romer and Jamieson, 2021; Kaspar and Nordmeyer, 2022). Our model proposed that persons holding a conspiratorial mindset distrust government health authorities, see them as having malign intentions and are drawn to media that promote misinformation and conspiracy theories about those actors (see also Pierre, 2020). In addition, the model predicted that persons with such a mindset avoid the mainstream media that tend not only to support vaccination but also to favorably report on health agency recommendations, such as masking (Romer and Jamieson, 2021, 2022).

Considerable research has examined the correlates of conspiratorial thinking (Stasielowicz, 2022; Hornsey et al., 2023), finding a small but positive relationship between dismissing randomness as an explanation for events and conspiracist ideation (Sternisko et al., 2022), and a pattern of anti-social tendencies, such as paranoia, and weakness in cognitive ability associated with it as well. One characteristic that stands out in regard to conspiratorial political beliefs is the tendency to distrust the intentions of those in power (Imhoff and Bruder, 2014), which is also a factor in our model. As also noted by Imhoff and Lamberty (2018), while those with a paranoid mindset tend to believe in a “threat from everyone to the self,” those with a conspiracy mindset “typically identify threat from only a small group of people affecting everybody.” Because governments and governmental agencies are run by powerful actors, they are likely to fall under the scrutiny of those with a conspiratorial mindset.

We showed that people holding the mindset a year before the pandemic not only doubted the credibility of government health authorities and the safety and efficacy of other vaccines at that time (such as the one to protect children from measles, mumps and rubella or MMR) but also were more likely a year after the pandemic arrived to accept misinformation about the safety and efficacy of COVID vaccines and to hold conspiracy beliefs about the pandemic and the vaccines developed to combat it. Surprisingly, those with the mindset appeared to grant comparable credibility to misinformation about COVID vaccines and conspiracy theories about them, suggesting that misinformation and conspiratorial thinking go hand in hand. In addition, as observed in other studies, persons accepting conspiracy theories about the pandemic were also more likely to downplay the seriousness of the health threat suggested by health authorities (Romer and Jamieson, 2020; McCarthy et al., 2022; van Prooijen et al., 2022).

A second component of the model was the prediction that those with the mindset are more likely to seek information from sources that support it. As predicted, they were more likely to rely on media that promoted conspiracy theories (Evanega et al., n.d.; The Annenberg IOD Collaborative, 2023) and that provided information congenial with them (Motta et al., 2020; Motta and Stecula, 2023). Consistent with their lack of trust in government and other power centers, these individuals also were more likely to avoid mainstream media that tended to advance the recommendations of governmental health authorities. In other words, the seeds of resistance to COVID vaccination and other preventative behaviors in the US were sown well before the arrival of the pandemic and were cultivated by media outlets that catered to those with conspiratorial mindsets.

Our prior test of the model focused on the first year of the pandemic just as vaccines for COVID became available. In this study, we tested the model’s ability to predict adult reluctance to vaccinate children ages 5–11 for COVID in the US much later in the pandemic. A recent cross-sectional study found that willingness to vaccinate children for COVID in the US before those vaccines were authorized for this age group was inversely related to belief in various vaccine conspiracies (Allen et al., 2023). A study in Italy found a similar result (Iannello et al., 2022). Using a panel in existence since April 2021, we assessed a general conspiratorial mindset at the first wave to see whether it would predict willingness to recommend giving a COVID-19 vaccine to a child ages 5–11 in January and March of 2022, several months after those vaccines were authorized for that age group. A prior study with this panel found that belief in misinformation about the safety and efficacy of vaccination was a strong predictor of support for child vaccination (Romer et al., 2022). However, that study did not examine the role of conspiratorial mindset as a factor underlying the acceptance of vaccination misinformation.

In this study, we hypothesized that holding a conspiratorial mindset almost a year earlier would predict lack of support for (a) vaccinating children against COVID-19 as well as against MMR as mediated by (b) acceptance of various forms of misinformation about vaccines in general, COVID-19 vaccines in particular, and conspiracy theories about those vaccines; (c) distrust of various healthcare agencies and providers; (d) belief in conspiracy theories about the pandemic apart from those specific to vaccines; and (e) perception that COVID-19 posed minimal risk to children as predicted by belief in the conspiracy theories in (d). We also hypothesized that (f) the mindset would predict use of media that promoted conspiracy theories and misinformation about vaccination while also predicting the avoidance of media that supported vaccination. In keeping with our earlier test of the model, we also expected that the mindset would negatively predict (g) the respondents’ uptake of COVID-19 vaccines for themselves as well as support for the long-established MMR vaccine, both of which would also be related to (h) reduced support for vaccinating children against COVID-19.

## Methods

### Survey sample

The study used data from a nationally representative probability sample drawn randomly from the SSRS Opinion Panel of U.S adults, 18 and older, and empaneled for this longitudinal study. As previously described (Romer et al., 2022), panel members were recruited randomly based on a nationally representative address-based-sample design (including Hawaii and Alaska). Additionally, hard-to-reach demographic groups were recruited *via* the SSRS Omnibus survey platform, a nationally representative (including Hawaii and Alaska) bilingual telephone survey designed to meet standards associated with custom research studies.

Panel members in our study were not selected for any other studies conducted by SSRS and are considered proprietary. Panelists were invited by email or telephone to participate in the panel and were compensated the equivalent of $15 for their time at each survey wave. The median length of the surveys was 20 min. The survey was deemed exempt from review by the Institutional Review Board of the University of Pennsylvania.

Of the 3,476 U.S. adult panelists invited to participate in wave 1 of the survey, 1,941 completed that wave’s survey in April 2021 (56% completion rate). The majority completed the survey online rather than by telephone (96% online and 4% by telephone) and in English rather than Spanish (98% and 2%, respectively). The original 1,941 panelists were re-contacted at each subsequent wave. Post-wave 1 panelist completion rates were high, averaging 85 percent each wave and have remained high in subsequent waves 2 through 6. The demographic distributions of the sample at wave 1 (April, 2021) and waves 5 (January, 2022) and 6 (March, 2022) show that the panel’s representation remained stable over time (see Table 1).

**Table 1:** Demographic distributions at wave 1 and waves 5 and 6 (unweighted).

Demographic characteristic	Wave		
	1	5	6
Age			
18–29	18.0	17.4	16.7
30–49	32.7	34.5	34.5
50–64	25.8	25.8	25.9
65+	23.4	22.3	22.9
Education			
No college	19.6	19.3	18.9
Some college	30.5	29.4	29.9
College or more	49.8	51.3	51.2
Household income			
Less than $50K	38.2	36.7	36.5
$50K but < $100K	34.0	34.4	34.6
$100K or more	27.3	28.4	28.3
Gender			
Male	48.3	49.2	49.2
Female	51.7	50.8	50.8
Party identification			
Democrat	47.8	47.6	47.7
Republican	33.1	33.7	33.7
Ind/Other/None	19.1	18.7	18.5
Evangelical christian	23.3	22.1	22.8
Parent of child < 18	25.9	26.7	26.6
Sample size	1941	1,656	1,638

### Measures

The baseline survey contained most of the items included in the study. Within that survey, assessment of the respondent’s COVID-19 vaccination status occurred first, followed by (a) questions about the trustworthiness of various health authorities, (b) knowledge regarding vaccination in general as well as toward COVID-19, (c) belief in conspiracy theories about COVID-19 vaccination and the pandemic, (d) conspiracy mindset items, (e) media use, and (f) demographic information. Respondent vaccination status was asked at the start of each subsequent wave. Shortly thereafter, questions about childhood COVID-19 vaccination were asked at the fifth and sixth waves, risk to children from COVID-19 was asked at the fifth wave, and willingness to have children receive the MMR vaccine was asked at the sixth wave. There were no skip patterns in any of the questioning except for the respondent’s vaccination status.

#### Vaccine misinformation

As shown in Table 2, we used a battery of 13 items that had been used in prior studies to assess beliefs about the safety and efficacy of vaccination (Jamieson et al., 2021; Romer et al., 2022; Romer and Jamieson, 2022). Each item was rated on a scale from *Definitely false* (1) to *Definitely true* (4). As we have done in prior studies, we coded the *Not sure* response in the middle of the scale (2.5). The table also includes item loadings determined in the measurement model described below.

**Table 2:** Vaccination misinformation and vaccine conspiracy theory (c) items and loadings on the misinformation factor (coded as reflecting greater misinformation).

Item	Loading
Vaccines in general are full of toxins and harmful ingredients like antifreeze	0.766
It’s safer to get the COVID-19 vaccine than to get COVID-19^a^	0.753
Vaccines give to children for diseases like measles, mumps, and rubella do NOT cause autism^a^	0.569
Increased vaccinations are why so many kids have autism these days	0.726
Allergic reactions to authorized vaccines against COVID-19 are very rare^a^	0.627
Getting a flu shot increases your risk of contracting COVID-19	0.686
COVID-19 vaccine changes people’s DNA	0.678
The pharmaceutical industry created the coronavirus to increase sales of its drugs and vaccines (c)	0.764
Vaccines approved for use in the U.S. are safe^a^	0.762
The Moderna and Pfizer COVID-19 vaccines contain fetal tissue	0.640
The vaccine against COVID-19 being developed with support by Microsoft founder Bill Gates contains microchips that can track the person who has been vaccinated (c)	0.765
COVID-19 vaccines are effective in preventing COVID-19^a^	0.747
Taking a COVID-19 vaccine can give you COVID-19	0.714

a Item was reverse scored to reflect misinformation.

#### Conspiracy mindset

As shown in Table 3, we used a combination of general conspiracy mindset items (labelled G) and items that referred to specific conspiracies (labelled S). Both were used in the prior test of the mindset model (Romer and Jamieson, 2022), and we confirmed that they loaded on the same factor.

**Table 3:** Conspiracy mindset items and loadings on mindset factor.

Item	Loading
(S1) Public water fluoridation is really just a secret way for chemical companies to dump the dangerous byproducts of phosphate mines into the environment	0.754
(S2) Certain U.S. government officials planned the attacks of September 11, 2001, because they wanted the United States to go to war in the Middle East	0.674
(S3) The FDA is deliberately preventing the public from getting natural cures for cancer and other diseases because of pressure from drug companies	0.794
(G1) Much of our lives is controlled by plots hatched in secret places	0.781
(G2) Even though we live in a democracy, a few people will always run things anyway	0.517
(G3) The people who really ‘run’ the country are not known to the voters	0.691

#### Pandemic conspiracy beliefs

We assessed belief in three conspiracy theories concerning the pandemic that we have used in prior research (Romer and Jamieson, 2020). These items shown in Table 4 were also rated on a scale from *Definitely false* (1) to *Definitely true* (4), with the *Not sure* response coded as 2.5.

**Table 4:** Conspiracy theory items and loadings on the pandemic conspiracy theory factor.

Item	Loading
Health officials at the Food and Drug Administration, also known as the FDA, who opposed Donald Trump’s re-election, delayed the approval of COVID-19 treatments until after the election	0.820
The coronavirus was created by the Chinese government as a biological weapon	0.682
Some health officials at the U.S. Centers for Disease Control and Prevention, also known as the CDC, exaggerated the danger posed by the coronavirus in order to damage the Trump presidency	0.853

#### Trust in health authorities

We assessed trust using four items from previous research (Romer and Jamieson, 2022) shown in Table 5. Respondents were asked: *In general, how confident are you that the source is providing you with trustworthy information about means of preventing and treating COVID-19.* Responses were recorded on a scale from *Not at all confident* (1) to *Very confident* (4).

**Table 5:** Trust in the health system items and their loadings on the trust factor.

Item	Loading
The doctor or nurse who is your primary health care provider is providing you with trustworthy information about means of preventing and treating COVID-19? (missing if no provider)	0.489
Dr. Anthony Fauci of the National Institutes of Health (NIH)	0.865
The Food and Drug Administration (FDA)	0.805
The U.S. Centers for Disease Control and Prevention (CDC)	0.896

#### Perceived risk of COVID to children

We assessed the seriousness of the health threat using two items designed for this research that concerned the consequences of not vaccinating a child (Table 6). Each item was rated on a scale from Not at all likely (1) to Very likely (4).

**Table 6:** Perceived risk of COVID items and their loadings on the risk factor.

Item	
How likely, if at all, are children ages 5 to 11 to be hospitalized with COVID-19 if they get COVID-19 and have not been vaccinated against it?	0.967
How likely, if at all, are children ages 5 to 11 to die of COVID-19 if they get COVID-19 and have not been vaccinated against it?	0.772

#### Sources of information in the media

Using a format similar to previous studies (Romer and Jamieson, 2021, 2022), we assessed various information sources by asking: *How often, if at all, do you get information about the most important issues of the day from…?* (Table 7). Responses were recorded on a scale from *Never* (1) to *All the time* (5). Sources such as Fox News and Newsmax were treated as either conservative or ultra-conservative in political viewpoint, while the major broadcast TV stations, major newspapers, and social media were considered less politically conservative. Univision and sources such as BET were treated as primarily serving a non-White audience.

**Table 7:** Measures of sources of information in the media.

Item
Sources such as Fox News
Sources such as Newsmax, One America News (OAN), Gateway Pundit, Parler or Telegram
Sources such as CBS News, NBC News, or ABC News
Sources such as the Associated Press, the news pages of the Wall Street Journal, or The New York Times
Sources such as Facebook, Instagram or Twitter
Univision
Sources such as Black Entertainment Television (BET), OWN, TV One, Bounce or The Root

#### Support for child vaccination

To assess this outcome, we used an item at both the fifth and sixth waves that we have analyzed in prior research: *If a child between the ages of 5 and 11 in your household were eligible to get the vaccine, how likely, if at all, would you be to recommend that child get vaccinated with the COVID-19 vaccine the FDA authorized?* Responses were recorded from *Not at all likely* (1) to *Very likely* (4). The items loaded equally high on a single factor (0.95).

#### Support for MMR vaccine

We also hypothesized that a conspiratorial mindset would be inversely related to support for other more established vaccines for children. Thus, at the sixth wave we asked respondents a hypothetical question about the MMR vaccine: *The US Centers for Disease Control and Prevention (CDC) recommends that children get a first dose of the measles, mumps, and rubella vaccine (MMR vaccine) at 12 to 15 months and a second dose when they are between ages 4 and 6 years old. If a child either between ages 12 to 15 months or between ages 4 and 6 years old in your household were eligible to get the vaccine, how likely, if at all, would you be to recommend that person get an MMR vaccine?* Responses were recorded from *Not at all likely* (1) to *Very likely* (4).

#### Vaccination for COVID-19

We have shown previously that respondents who have received the recommended doses of COVID vaccine were more likely to recommend the vaccine for children. As we have found in prior research, the conspiratorial mindset would also be expected to predict vaccination for oneself. Thus, we included an assessment of the respondent’s vaccination status at the sixth wave. Respondents were asked [1] if they had been vaccinated, [2] if so, which vaccine had they received, and [3], if Moderna or Pfizer, had they received the second dose. Individuals were coded as having completed the primary series if they received the single dose of the Johnson & Johnson vaccine or both primary series doses of either the mRNA vaccines (Moderna or Pfizer). Receipt of the recommended dosage was coded as 1 and not as 0. At the sixth wave, approximately 69% of the panel reported having received the primary series, which is similar to the rate reported by CDC (66%, USA Facts, 2023).

### Analysis

We used confirmatory factor analysis implemented in Mplus (Muthén and Muthén, 1998) to identify a measurement model for the factors hypothesized to be related to vaccination intentions and behavior. After confirming the measurement model, we tested the conspiratorial mindset model using a structural equation model (SEM) based on Romer and Jamieson (2022). The model held constant the various demographic differences in Table 1 as covariates of conspiratorial mindset. We used full information maximum likelihood to impute missing cases as implemented in Mplus. We used bootstrapping to determine 99% and 95% confidence intervals for all parameters in the model. We dropped relations that were below a threshold of 90% confidence, and we report standardized coefficients for the model parameters.

## Results

### Conspiratorial mindset

**Figure 1:**
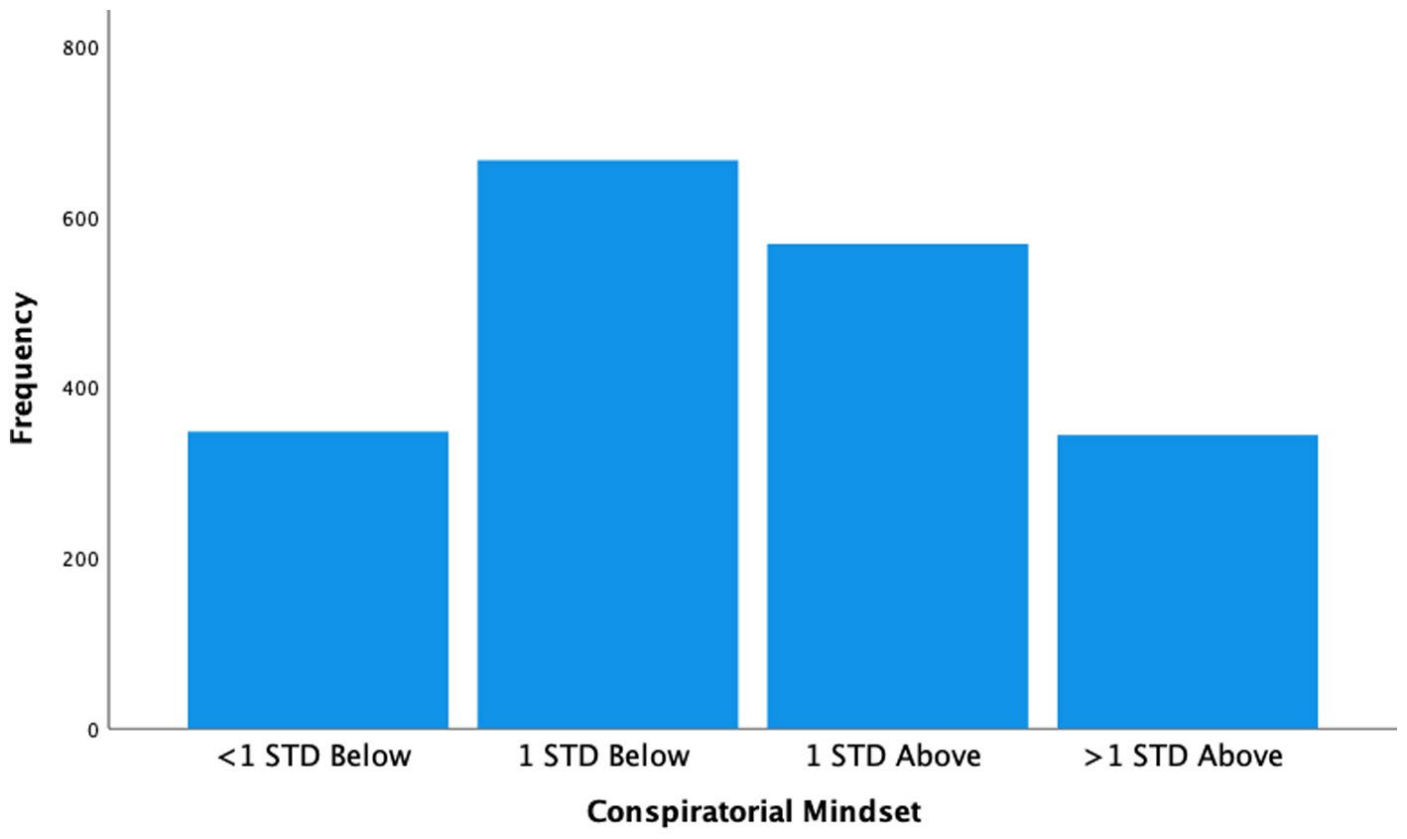
Distribution of conspiratorial mindset with approximately 17% of the sample more than 1 standard deviation above the mean (*N* = 1926).

The distribution of the mindset scale based on the first principal component (shown in Figure 1) distinguishes those who scored above and below 1 standard deviation from the mean. Similar to what we have observed in our prior studies (Romer and Jamieson, 2021; Romer and Jamieson, 2022), those scoring 1 standard deviation above the mean represented about 17% of the sample.

### Measurement model for SEM

The measurement model provided a good fit to the data with low RMSEA (0.050, 90% CI = 0.048, 0.052), high CFI (0.942), and low SRMR (0.039). The intercorrelations between the factors in the model and vaccination measures are in Table 8. Recommendation to vaccinate a child for COVID was highly related to belief in misinformation, trust in the health system, and less so for perceived risk of COVID for children. As expected, conspiratorial mindset and belief in COVID conspiracies were negatively related to recommendations to vaccinate for COVID. In addition, the correlation between willingness to vaccinate children for either MMR or COVID was high (0.542), and both were related to the vaccination status of the respondent, reflecting the overall lack of support for vaccination.

**Table 8:** Intercorrelations between factors in the measurement model and measures of vaccination.

Factor	A	B	C	D	E	F	G
(A) Misinformation							
(B) Trust	−0.690						
(C) Risk to child	−0.265	0.410					
(D) Conspiratorial mindset	0.888	−0.652	−0.187				
(E) COVID conspiracies	0.752	−0.741	−0.346	0.745			
(F) Vaccinate child	−0.721	0.777	0.545	−0.615	−0.709		
(G) Vaccinate for MMR	−0.569	0.465	0.216	−0.464	−0.428	0.542	
(H) Vaccinate self	−0.569	0.268	0.189	−0.341	−0.252	0.359	0.222

There were also a few correlations between items that were related beyond the factors on which they loaded. For example, there was an additional relation between the belief that vaccines in the US are safe and the trust factor (0.092) and between the belief that Bill Gates was behind the vaccination program and the COVID conspiracy factor (0.047). There were also additional relations between the conspiracy mindset item regarding fluoridation and belief that failing to vaccinate a child for COVID could lead to hospitalization (0.432) and death (0.193). These residual relations were also observed for recommendations to vaccinate a child for COVID (0.160 and 0.185). Apparently, belief in the fluoridation conspiracy also had a positive relation with belief in the risk of COVID. Nevertheless, the item loaded heavily on the overall conspiracy mindset factor, indicating that most of its influence was negative regarding vaccination.

### Predictors of childhood vaccination

**Figure 2:**
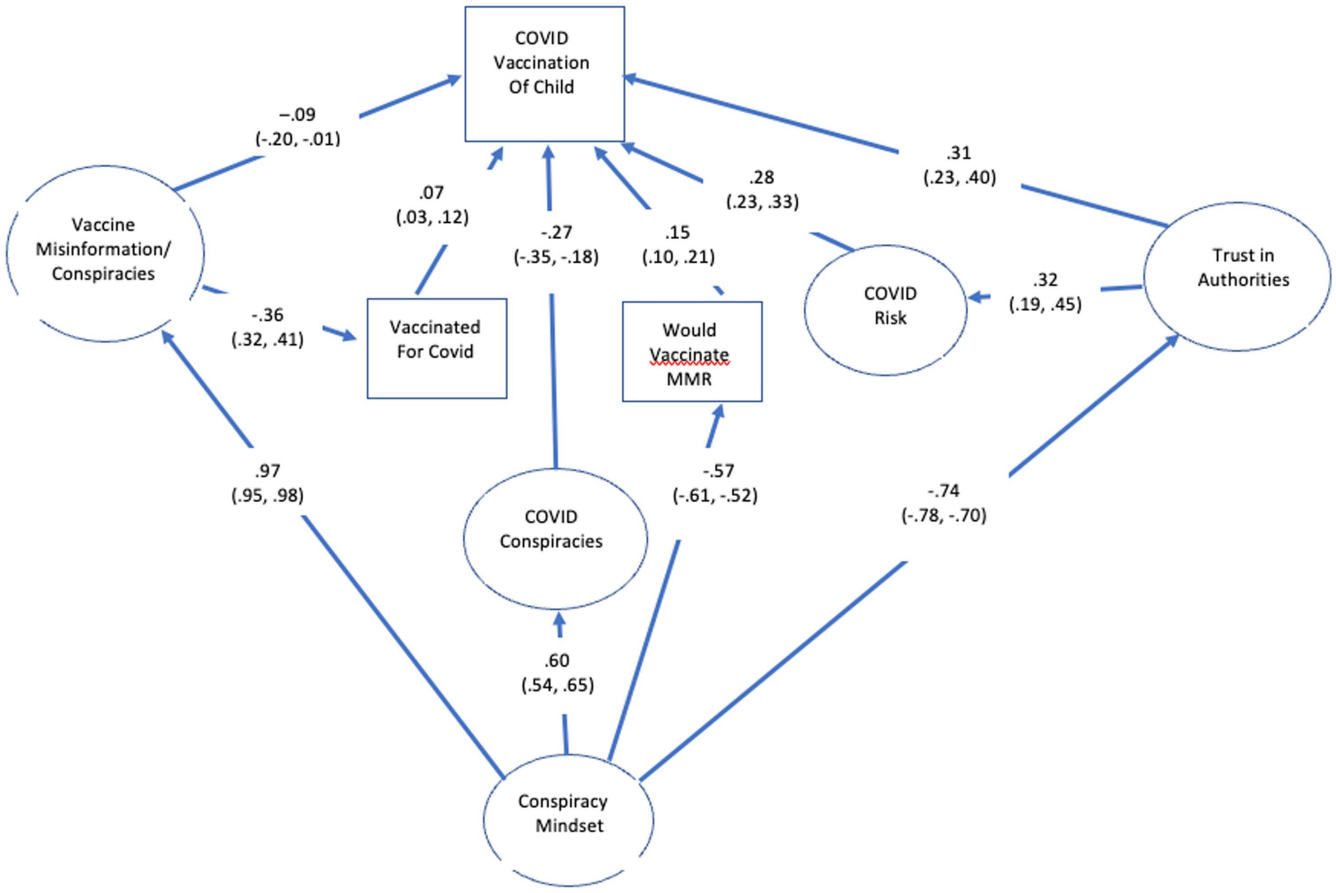
SEM results for major predictors of child vaccination.

The major predictors in the SEM are shown in Figure 2. The model provided a good fit to the data: low RMSEA (0.044, 90% CI = 0.043, 0.045), high CFI (0.917), and low SRMR (0.047). In addition, the model accounted for 76% of the variance in child vaccination for COVID. We first describe the relations between the mindset and the direct predictors of vaccination [hypotheses (a) to (d) and (f) and (g)] and then focus on the predictors of media use [hypothesis (e)]. Consistent with the prediction that conspiracy mindset would underlie all of the more direct predictors of COVID vaccination, it was positively related to belief in COVID conspiracies (0.60, 99% CI = 0.54, 0.65), belief in misinformation about COVID vaccines (0.97, 99% CI = 0.95, 0.98) and negatively related to trust in the health system (−0.74, 99% CI = -0.78, −0.70) and willingness to vaccinate a child for MMR (−0.57, 99% CI = -0.61, −0.52). While the mindset factor was not directly related to the respondent’s COVID vaccination status, it was indirectly related *via* vaccine misinformation (0.97 X − 0.36 = −0.35).

The largest single predictor of recommending a COVID vaccination for a child was trust in health authorities (0.31, 99% CI = 0.23, 0.40). Consistent with what was found in the previous test of the conspiracy mindset model, trust in health authorities also had an indirect relation with recommendation to vaccinate against COVID *via* the perceived risk of COVID (0.32, 99% CI = 0.19, 0.45), which in this case referred to risk to unvaccinated children.

As expected, respondents’ willingness to accept a vaccination for themselves for COVID (0.07, 99% CI = 0.03, 0.12) and for MMR in children (0.15, 99% CI = 0.10, 0.21) were positively related to recommendations to vaccinate a child against COVID-19. In addition, belief in misinformation about COVID and other vaccines was negatively related to recommendations to vaccinate a child for COVID both directly (−0.09, 95% CI = -0.20, −0.01) and indirectly *via* vaccination for oneself (−0.09 × 0.07 = −0.01) for a total of −0.10.

**Figure 3:**
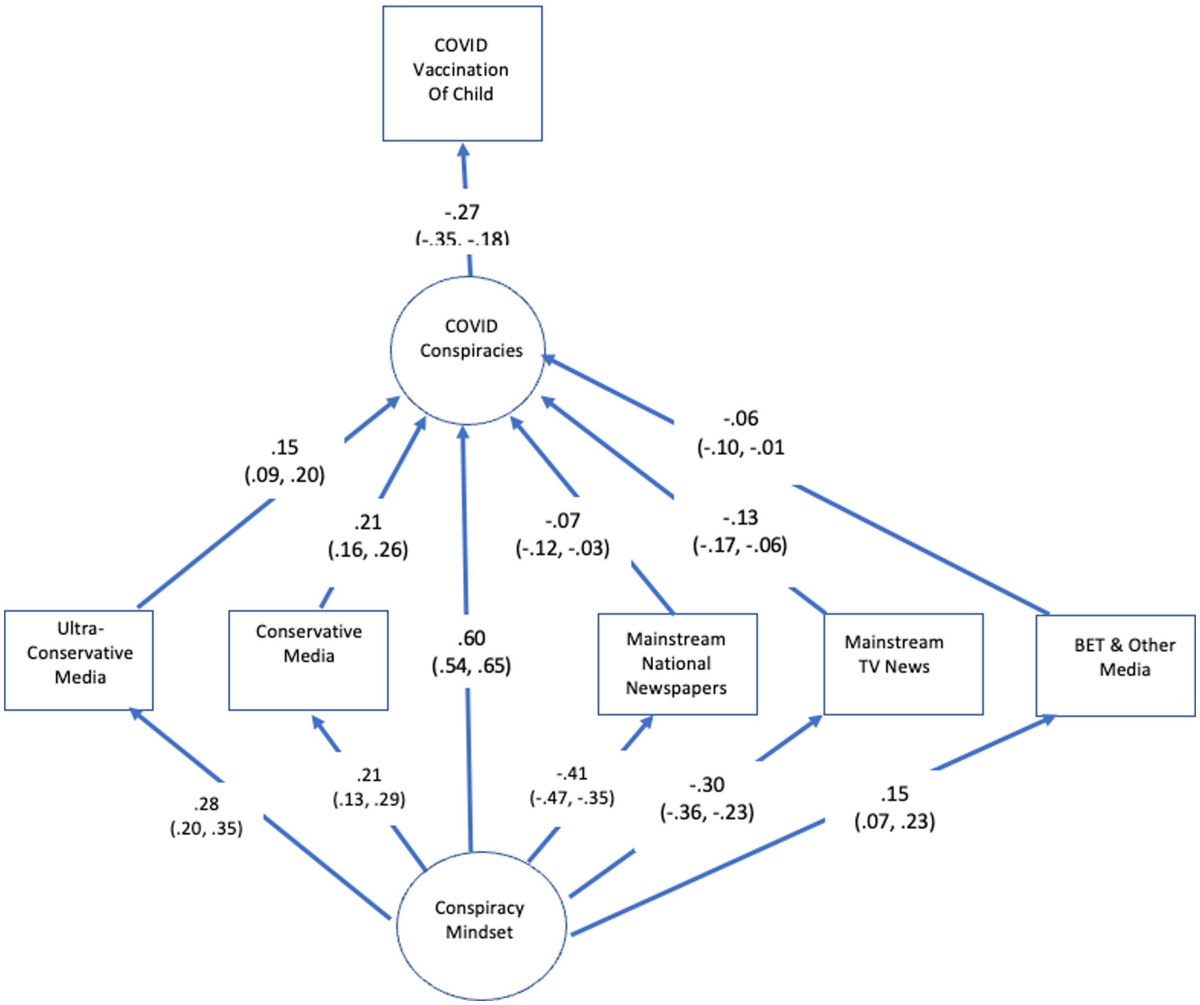
SEM results for relationship between the mindset and media.

The conspiracy mindset model also made predictions about exposure to media that either supported or undermined vaccination *via* belief in conspiracy theories about the pandemic. As seen in Figure 3, those with the mindset were more likely to use Fox News and ultra-conservative media, and those media were associated with belief in COVID conspiracy theories. At the same time, those with the mindset were less likely to use more mainstream sources, such as national newspapers and TV news, both of which were associated with less belief in COVID conspiracy theories. Finally, those with the mindset were slightly more likely to also use BET and other media directed to non-white audiences, but these media were negatively related to belief in COVID conspiracies.

Overall, by virtue of differences in media use, holding the mindset was positively related to believing in conspiracy theories about the pandemic (0.14, 99% CI = 0.11, 0.18), which was associated with less interest in recommending the vaccine for children.

We also tested for relations between belief in pandemic conspiracy theories and other uses of media, but no other ones directly predicted that outcome holding constant the ones in Figure 3. For example, use of social media and Univision did not directly predict belief in pandemic conspiracy theories.

Aside from the direct predictors of child vaccination in Figure 2, there was still one demographic characteristic that predicted the outcome: evangelical religious identity (−0.043, 99% CI = −0.088, −0.002). This characteristic also predicted some uses of media. For example, evangelical respondents were more likely to use Fox News (0.08, 99% CI = 0.02, 0.15) and BET (0.11, 99% CI = 0.03, 0.22). They were also less likely to use national newspapers (−0.07, 99% CI = −0.13, −0.02). However, uses of those media could not totally account for the lack of support among evangelical respondents for child vaccination. This pattern of results indicates that the factors in the model fully accounted for the relation between childhood vaccine for COVID and all demographic differences in the model except for evangelical identity.

Other demographic differences had direct relations with perceived risk of COVID and conspiracy beliefs about the pandemic. In particular, both Black (0.11, 99% CI = 0.03, 0.19) and Hispanic (0.35, 99% CI = 0.09, 0.63) respondents reported more perceived risk of COVID-19 to children, while male respondents perceived less (−0.12, 99% CI = -0.20, −0.06). Viewers of national TV news were also more likely to believe that COVID presented a risk to children (0.22, 99% CI = 0.14, 0.29).

Respondents who identified as Republican (0.21, 99% CI = 0.14, 0.27) and Independent (0.059, 99% CI = 0.004, 0.112) were more likely to express belief in pandemic conspiracy theories, which translated into less support for COVID-19 vaccination.

As observed in the previous test of the model, there were positive correlations between the various conspiracy mindset items representing measurement error that reflected the social undesirability of expressing belief in the theories (see also Smallpage et al., 2022). Removing these correlations served to increase relations between the mindset factor and its various outcomes in the model.

Also not shown in Figures 2, 3 are the various demographic characteristics that were related to conspiratorial thinking and held constant in testing the relation between the mindset and other factors. In particular, Black (0.16, 99% CI = 0.09, 0.21), Hispanic (0.13, 99% CI = 0.07, 0.19), evangelical (0.20, 99% CI = 0.14, 0.26), or Republican (0.16, 99% CI = 0.10, 0.22) respondents as well as parents with children less than 18 years of age (0.22, 99% CI = 0.16, 0.27) were more likely to have conspiratorial mindsets. On the other hand, respondents with higher income (−0.30, 99% CI = −0.36, −0.25), or with more education (−0.37, 99% CI = −0.42, −0.31) or who were older (−0.16, 99% CI = −0.22, −0.10) were less likely to have those mindsets. We did not include a separate predictor for parents with children in the 5–11 age bracket because they overlapped substantially with those who had children under age 18 and their prediction did not differ from that of the larger group of parents.

## Discussion

Our test of the conspiratorial mindset model of vaccination largely replicated what we observed in our prior test of the model (Romer and Jamieson, 2022). The present findings with a more recent national panel indicated that a discernible segment of the US population (about 17%) held a conspiratorial mindset toward the government in general and toward US health agencies in particular.

We again found that those holding a conspiratorial mindset were more likely to believe various forms of misinformation about the safety and efficacy of vaccination in general and for COVID in particular. These beliefs also included conspiracy theories about the role of the pharmaceutical industry and Bill Gates in promoting vaccines in a secretive and harmful manner. As observed in the prior study, beliefs in conspiracy theories about vaccines were highly related to misinformation about vaccines, suggesting that these beliefs go hand in hand in the minds of conspiracy believers. High correlations between misinformation and conspiracy theories about the pandemic have been observed across multiple countries (De Connick et al., 2021).

The mindset also was directly related to belief in various conspiracy theories about the pandemic, such as that the Chinese had developed the virus as a biological weapon or that elements within the health agencies had delayed the development of the vaccine to undercut Donald Trump’s re-election prospects. These beliefs were popularized during the 2020 election (Romer and Jamieson, 2020) and continued to hold sway among those with the mindset at the time of the baseline survey. Nevertheless, belief in conspiracies about the causes and response to the pandemic formed a separate factor from conspiracies about vaccines *per se*, indicating that the mindset does make distinctions between different conspiracy theories (see also Goertzel, 1994; Davis et al., 2018). Belief in theories about the causes of the pandemic tended to be associated with less concern about the health effects of the crisis while belief in vaccine conspiracies tended to be more directly associated with resistance to vaccine uptake. In either case however, the conspiratorial mindset as mediated by belief in specific conspiracy theories was negatively related to willingness to vaccinate both the respondent as observed in the prior Romer and Jamieson study (2022) and children in general.

Consistent with the mindset model, conspiratorial thinking was also inversely related to trust in various health authorities, including one’s own health care provider. In addition, because trust was directly related to the perceived risk that the virus posed to children, that negative relation with trust was also associated with less support for child vaccination among those with the mindset. These findings are consistent with the longstanding observations that holding conspiratorial beliefs is a barrier to engaging in various health-promoting behaviors, such as HIV prevention (Bogart and Thorbun, 2005), use of recommended medications (Oliver and Wood, 2014), and other government recommended behavior during the COVID pandemic (Romer and Jamieson, 2020; Pellegrini et al., 2021). It also is consistent with concerns that trust in health agencies is a barrier to obtaining widespread uptake of COVID vaccines (Pertwee et al., 2022).

### The role of the media

The conspiratorial mindset model also predicted that those holding such a mindset would be attracted to media that promoted conspiracy theories and would avoid media that did not do so. Here again, we found support for this prediction. Those with the mindset were more likely to use conservative media, which in the US have promoted various conspiracies and misinformation about the pandemic (Evanega et al., n.d.; Motta et al., 2020; Savillo and Monroe, 2021) and featured conspiracy theories alleging that the 2020 election was stolen from the former president (Tollefson, 2021; The Annenberg IOD Collaborative, 2023). As noted above, those with the mindset also were more likely to hold conspiracy theories regarding the 2020 presidential election and these beliefs were associated with use of conservative media. At the same time, those with the mindset tended to avoid the media that did not support those conspiracies, such as mainstream newspapers and TV news. Perhaps not surprisingly, those with the mindset were more likely to follow BET and other media directed to non-white Americans. Nevertheless, those media were negatively related to belief in pandemic conspiracies. Thus, for those with that mindset, especially Black respondents, their media use had a small negative relation with belief in specific pandemic conspiracies.

While our model is similar to the one proposed by Pierre (2020) for the role of conspiratorial thinking in distrusting government authorities and seeking media that confirms that distrust, it also predicts that the mindset will lead to avoidance of information sources such as mainstream TV and newspapers that are generally more aligned with the views of federal public health agencies. Those sources have been famously called “fake news” by the former president who was a promoter of several conspiracy theories about the workings of the government (Norris et al., 2020; Tollefson, 2021; The Annenberg IOD Collaborative, 2023). It appears that those with the mindset are drawn to information that supports their distrust of authority and avoid sources that do not reinforce these views. As a result, they are likely to be engulfed in an echo chamber, at least in regard to issues surrounding the government and its role in protecting public health (Romer and Jamieson, 2022).

This study adds importantly to our prior findings in the following ways. First, in it, we focused on reported adult vaccination rather than intent to vaccinate. Second, here we found that conspiratorial thinking is implicated in the lack of support for two different childhood vaccines: those for COVID-19 and for MMR. The conspiratorial mindset has also been related to delay in vaccination for HPV (Callaghan et al., 2019) and to reduced willingness to take the adult flu vaccine (Romer and Jamieson, 2022). The findings presented here add further evidence that conspiratorial thinking is implicated in the lack of support for a wide range of vaccines. Finally, by replicating what we found in our prior study in a different national survey conducted before COVID vaccines were available in the US, this study shows the robustness of the model across time and samples.

### Other motives for conspiracy mindset

Our model of conspiracy mindset proposes two major motivations for this tendency: first, a predisposition to see those in power as posing a threat to the public, leading to questioning the credibility and intentions of health authorities, the government, and mainstream media. Second, the search for information that confirms conspiratorial explanations for events that threaten the wellbeing of the public. This motive also leads to the discrediting and avoidance of information sources that represent the interests of those in power.

We can contrast this model with others that have focused on various motives for conspiracy [reviewed by Douglas et al. (2019) and Hornsey et al. (2023)], some of which differ from our model. Among these, the epistemic model claims that persons drawn to conspiracy theories are merely seeking explanations for events that are novel or difficult to explain (Douglas et al., 2019). However, the robustness of our media use findings is at variance with that explanation. For example, a purely epistemic motive would not predict avoidance of particular news and opinion sources, which are more likely to support government recommendations and explanations for events such as pandemics. Such selective exposure suggests a more biased information-search strategy. Two studies in Italy found evidence for unbiased epistemic motives by asking whether the pandemic was an expected event (Giacomantonio et al., 2022). Those who said it was also reported less belief in pandemic conspiracy theories, suggesting that the need to explain an unexpected event motivated the acceptance of pandemic conspiracy theories. However, it is just as possible that those without a conspiratorial mindset accepted other explanations for the pandemic that were more in keeping with what health authorities advocated. What may have distinguished those with the mindset was the belief that the pandemic was unusual due to the actions of powerful and malevolent agents. In sum, the need for an explanation for an event does not seem to distinguish those with the mindset from others who adopt non-conspiratorial explanations for those events.

Another epistemic explanation for the adoption of conspiracy theories is the need for cognitive closure, a need defined as individuals’ “desire for a firm answer to a question, any firm answer as compared to confusion and/or ambiguity” (Kruglanski, 2004). This individual difference has been found to predict acceptance of conspiracy theories for events that lack a clear explanation (Marchlewska et al., 2018). While this tendency may predict adoption of conspiracy theories in specific cases, it appears to be unrelated to the mindset (Leman and Cinnirella, 2013; Imhoff and Bruder, 2014), which is a tendency to doubt the credibility of explanations emanating from those in power. The need for closure also predicts acceptance of any explanation that is salient at the moment (Leman and Cinnirella, 2013), whether it originates from those in power or not. Thus, it may be a predisposition to accept firm answers in the presence of ambiguity whether the answers are conspiratorial or not.

It has also been proposed that belief in conspiracy theories serves social motives by enabling a positive self-image. But this would not explain why persons with the mindset are drawn specifically to conspiracies. There is evidence that those endorsing the mindset see themselves as lacking political influence (Goertzel, 1994; Abalakina-Paap et al., 1999; Bruder et al., 2013). For some, such as Black Americans (Goertzel, 1994; Davis et al., 2018), the mindset accords with their experiences of abuse at the hands of the government. These sources of discontent may lead to adopting the mindset, but seeking a positive self-image does not appear to distinguish those with the mindset from others.

A third motive termed existential refers to a variety of threats that encourage belief in conspiracy theories. However, what constitutes an existential threat is less clear. There is evidence that persons with the mindset feel alienated from the political system as noted above. This lack of political control may provide a partial explanation for the mindset. Consistent with this explanation, giving people a greater sense of control has been found to produce a short-term decline in conspiracy mentality (van Prooijen and Acker, 2015; Whitson et al., 2019).

At the same time, a different type of existential threat that reflects a sense of insecurity and danger has also been entertained. However, when this motive is assessed, it appears to increase following increases in belief in conspiracy theories, whereas increases in this threat do not appear to be followed by increases in conspiracy beliefs (Liekefett et al., 2023). Thus, feelings of insecurity do not appear to be a unique precursor to the mindset. For example, it is likely that many people felt threatened by the pandemic but accepted the guidance provided by governments to cope with the threat. It was those with the mindset who distrusted the government who were drawn to conspiratorial explanations for the pandemic. In sum, feelings of alienation and lack of control in the political context may well be a source of distrust and attribution of malevolent intentions toward government, mainstream media, and other elites that results in a conspiratorial mindset.

### Correlates of conspiratorial mindset

Several demographic characteristics were related to the mindset. As has been observed before, these relations suggest that the mindset is more common among the less educated and those with lower incomes (Goldberg and Richey, 2020; Casara et al., 2022). These individuals are more likely to feel left out of the economy. In addition, members of non-White communities were more likely to hold the mindset, potentially also reflecting prior exclusion from the economy and discriminatory treatment by the health system (Penner et al., 2007; Quinn et al., 2017; Brondolo et al., 2023; Cope et al., 2023). It is also not a surprise that those with a more conservative political view were more likely to evince the mindset, especially since the president and prominent voices in conservative media promoted conspiracy theories about a range of issues (Norris et al., 2020; Jamieson et al., 2021; Tollefson, 2021; The Annenberg IOD Collaborative, 2023).

### The role of misinformation

It was perhaps surprising that the overall relation between misinformation and support for vaccinating children for COVID was quite small. This was in contrast to an earlier study involving the same panel in which we found that belief in misinformation was a very strong predictor of this outcome (Romer et al., 2022). It is important to recognize however that in this study, misinformation was highly related to the other predictors in the model as driven by conspiratorial thinking. For example, misinformation and trust in health authorities were correlated 0.97 X − 0.74 = −0.78 due to shared variation with the mindset. Thus, without conspiratorial mindset in the model, misinformation would be a stronger predictor of support for child vaccination.

Despite the confound with conspiratorial mindset, misinformation is likely to be a powerful factor affecting views about vaccination (Loomba et al., 2021). However, the mechanism for bringing this about may rely on the source of the corrective information. For example, in a recent study, we found that changes in misinformation beliefs among Black respondents over the course of the vaccine rollout were predictive of changes in vaccination for this population (Romer et al., 2023). Some have attributed this change to pro-vaccination efforts by credible sources such as the Black clergy who encouraged their worshippers to overcome their conspiratorial thinking about the health system and accept the vaccine (e.g., Moore et al., 2022).

There also has been evidence that when statements by former president Trump were sent to hesitant communities *via* social media, those regions were more likely to take up the COVID vaccine (Larsen et al., 2022). In addition, when Republicans were shown a message from former president Trump supporting COVID vaccination, their intentions to receive the vaccine increased (Pink et al., 2021). Thus, it is possible to overcome or at least sidestep the effects of the mindset on vaccination when credible sources support vaccination. Unfortunately, many with the mindset use media that continue to support conspiratorial thinking about issues such as COVID vaccination, a reliance likely to reinforce belief in misinformation about vaccination in general and COVID in particular (Motta and Stecula, 2023).

#### Limitations

Our panel members tend to be more educated than typical Americans and thus their attitudes may not be completely generalizable to the entire population. In addition, our panel may have become sensitized to questions about vaccination. Nevertheless, our adult vaccination level was comparable to CDC’s administration data, and we controlled for education in the SEM. Reports of behavior and intentions are subject to social desirability biases, but our findings indicate that those with a conspiratorial mindset are willing to report lack of support for vaccination, suggesting that such biases are not a strong factor in our results. Furthermore, we found and removed correlations between the mindset items that reflected the social undesirability of expressing support for conspiracy theories, which should increase the validity of relations with the mindset factor.

## Conclusion

Holding a conspiracy mindset remains a powerful factor underlying adult reluctance to be vaccinated against COVID-19 and to recommend such vaccination for children ages 5–11 in the US. Consistent with the prior test of the mindset model, here we find that it is related to the major predictors of vaccination, and especially to belief in misinformation about the safety and efficacy of vaccines, belief in conspiracy theories about vaccines and the pandemic, and to trust in health care authorities. Increasing vaccination of children as well as adults among those with a conspiracy mindset may require credible sources able to disassociate the mindset from particular vaccines.

## Data availability statement

The raw data supporting the conclusions of this article will be made available by the authors, without undue reservation, the dataset is available at the following: https://osf.io/pm7w2/files/osfstorage/6456a9f40637da0a38a2d6da.

## Ethics statement

The studies involving human participants were reviewed and approved by Institutional Review Board of the University of Pennsylvania. Written informed consent for participation was not required for this study in accordance with the national legislation and the institutional requirements.

## Author contributions

DR and KJ conceptualized the study and wrote the report. DR conducted the analyses. All authors contributed to the article and approved the submitted version.

## Funding

Robert Wood Johnson Foundation supported the data collection but had no role in the analysis and writing of the paper.

## Conflict of interest

The authors declare that the research was conducted in the absence of any commercial or financial relationships that could be construed as a potential conflict of interest.

## Publisher’s note

All claims expressed in this article are solely those of the authors and do not necessarily represent those of their affiliated organizations, or those of the publisher, the editors and the reviewers. Any product that may be evaluated in this article, or claim that may be made by its manufacturer, is not guaranteed or endorsed by the publisher.
